# Surface charge manipulation and electrostatic immobilization of synaptosomes for super-resolution imaging: a study on tau compartmentalization

**DOI:** 10.1038/s41598-021-98142-1

**Published:** 2021-09-20

**Authors:** Ushashi Bhattacharya, Jia-Fong Jhou, Yi-Fong Zou, Gerald Abrigo, Shu-Wei Lin, Yun-Hsuan Chen, Fan-Ching Chien, Hwan-Ching Tai

**Affiliations:** 1 Department of Chemistry, National Taiwan University, Taipei, 106 Taiwan; 2 Department of Optics and Photonics, National Central University, Taoyuan, Taiwan

**Keywords:** Imaging, Molecular neuroscience, Synaptic plasticity

## Abstract

Synaptosomes are subcellular fractions prepared from brain tissues that are enriched in synaptic terminals, widely used for the study of neural transmission and synaptic dysfunction. Immunofluorescence imaging is increasingly applied to synaptosomes to investigate protein localization. However, conventional methods for imaging synaptosomes over glass coverslips suffer from formaldehyde-induced aggregation. Here, we developed a facile strategy to capture and image synaptosomes without aggregation artefacts. First, ethylene glycol bis(succinimidyl succinate) (EGS) is chosen as the chemical fixative to replace formaldehyde. EGS/glycine treatment makes the zeta potential of synaptosomes more negative. Second, we modified glass coverslips with 3-aminopropyltriethoxysilane (APTES) to impart positive charges. EGS-fixed synaptosomes spontaneously attach to modified glasses via electrostatic attraction while maintaining good dispersion. Individual synaptic terminals are imaged by conventional fluorescence microscopy or by super-resolution techniques such as direct stochastic optical reconstruction microscopy (dSTORM). We examined tau protein by two-color and three-color dSTORM to understand its spatial distribution within mouse cortical synapses, observing tau colocalization with synaptic vesicles as well postsynaptic densities.

## Introduction

Neurons communicate with the neighboring neurons by transmitting signals at specialized connections called synapses. The functional plasticity of synapses underlies learning, memory, and network computations^1–3^. The complexity of the brain could be partly explained by the astronomical number of synapses it contains. The human neocortex alone is estimated to contain 164 trillion synapses^4^. A very useful technique for studying synaptic terminals was to isolate them from homogenized brain tissues via subcellular fractionation. These biochemical fractions enriched in intact synaptic terminals (broken-off nerve endings) were generally termed “synaptosomes”^5,6^. Freshly prepared synaptosomes are metabolically active and capable of releasing neurotransmitters upon stimulation. Biochemical studies of synaptosomes provided a fundamental understanding of the mechanisms of neural transmission^7,8^. Synaptosomes have also been successfully prepared from post-mortem human tissues, and they were useful for the study of synaptic pathologies that underlie neurological disorders, especially Alzheimer’s disease (AD)^9–12^.

The study of protein localization and compartmentalization is very important for understanding synaptic function and dysfunction. Of particular interest is the involvement of local protein synthesis in synaptic plasticity^13,14^. Recently, immunofluorescence techniques have been increasingly applied to investigate synaptosomes for this purpose^12,15–20^. There are two advantages associated with imaging isolated brain synaptosomes compared to the immunohistochemistry of brain tissue slices: better antibody penetration and the elimination of signal interference from axonal and dendritic shafts and glial terminals. This is especially important when the target protein only has a minor fraction situated inside synapses. For instance, tau is an abundant marker protein of axons in healthy, mature neurons^21,22^, but imaging isolated brain synaptosomes enabled the discovery of postsynaptic tau by eliminating axonal signal interference^12^. The application of super-resolution fluorescence microscopy and/or expansion microscopy can further enhance the spatial resolution of synaptosome imaging^23,24^.

For the immunolabeling of intracellular proteins in synaptosomes, chemical fixation and detergent permeabilization are required for antibody penetration. The standard fixative for synaptosome immunofluorescence is formaldehyde—usually prepared from paraformaldehyde (PFA), which also helps crosslink synaptosomes to glass surfaces coated with proteins or polyamines^15,17,18^. However, we noticed aggregation artefacts caused by formaldehyde fixation in our previous studies, observing both clumped and dispersed synaptosomes over glass coverslips^12,16^. Synaptosomes that remained dispersed could be classified as the stand-alone presynapse, stand-alone postsynapse, or bipartite synapse (with presynaptic markers adjacent to postsynaptic markers)^10^. However, one could not differentiate whether the observed bipartite synapse was truly an intact synaptic terminal or a crosslinking artefact between a presynapse and a postsynapse. Clumping could also introduce an inherent statistical bias for stereological quantification, because we did not know if certain synapse subtypes were more prone to aggregate. Hence, there is a need to develop synaptosome preparations free of aggregation artefacts for optimal imaging conditions.

The primary disadvantage of formaldehyde fixation is its reversibility. Formaldehyde crosslinking consists of a series of reversible nucleophilic reactions, which cannot be effectively quenched^25,26^. The ongoing crosslinking reactions may eventually lead to covalent linkages between the surface proteins of individual synaptosome particles, leading to aggregation artefacts. Here, we seek to circumvent this issue by choosing the irreversible chemical fixative ethylene glycol bis(succinimidyl succinate) (EGS), an amine-reactive crosslinker with two N-hydroxysuccinimide (NHS) esters^27^. We devised a simple strategy to immobilize EGS-fixed synaptosomes over glass coverslips via electrostatic attraction while maintaining good dispersion. As proof-of-principle, we successfully imaged tau immunofluorescence in mouse cortical synaptosomes. Misfolded aggregates of tau protein and beta-amyloid (Aβ) peptide are the pathological hallmarks of AD, which accounts for ~ 50% of senile dementia cases^28,29^. Tau oligomers accumulating at synaptic sites may be responsible for the transmission of proteotoxicity in AD via a prion-like mechanism^30–33^. However, relatively little is known about the normal role of tau at synaptic sites before the onset of neurodegeneration^21,34–36^. Using direct stochastic optical reconstruction microscopy (dSTORM)^37^, we successfully identified tau colocalization with synaptic vesicles at the presynapse and with postsynaptic densities (PSD) at the postsynapse in wild-type mouse brains.

## Results

### EGS fixation does not cause aggregation

**Figure 1:**
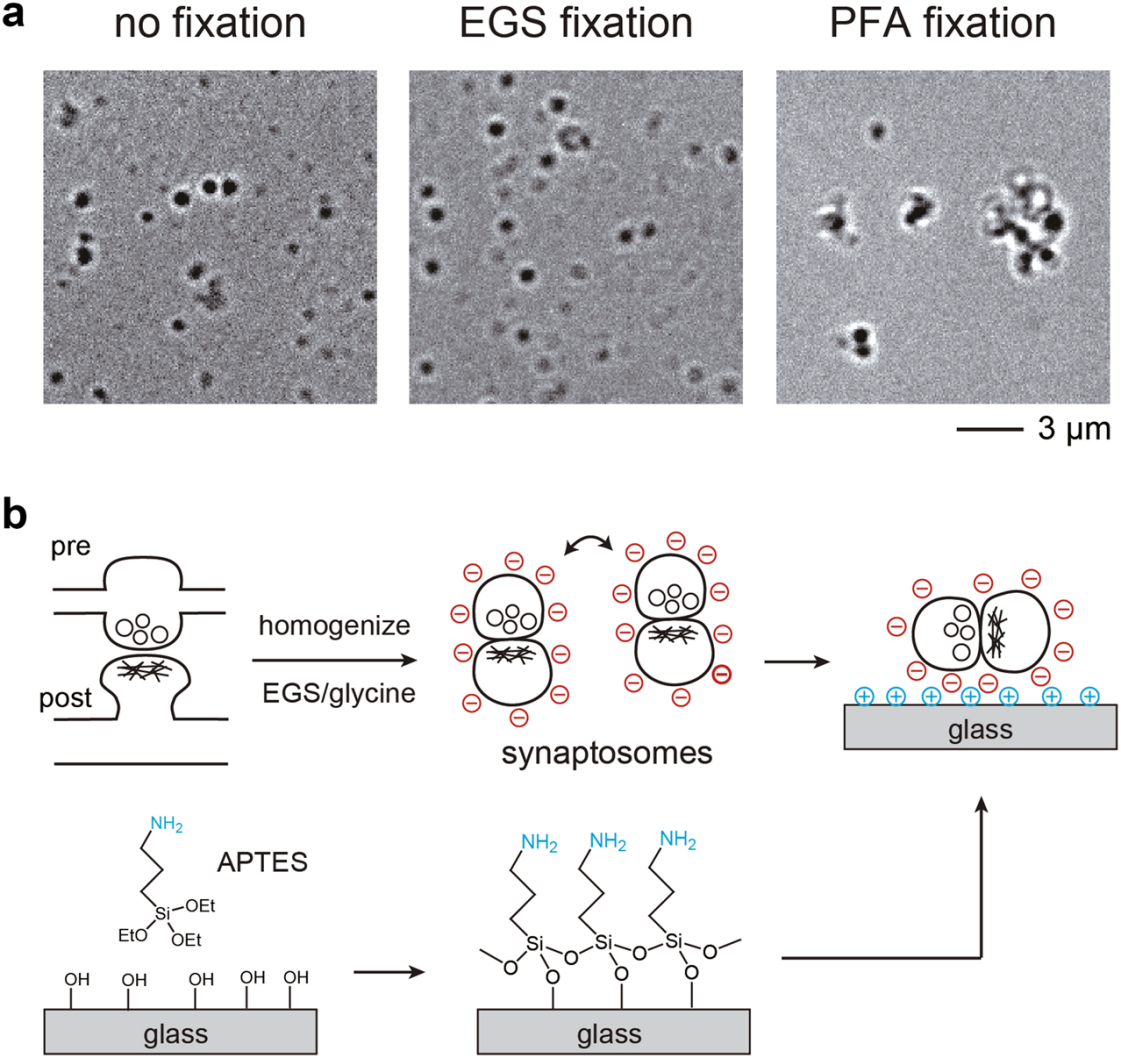
(**a**) Brightfield images showing non-fixed, 2 mM EGS-fixed, and 1% PFA-fixed synaptosomes captured over APTES-functionalized coverglass. (**b**) Schematics for the isolation of synaptosomes from brain tissues, followed by EGS fixation and attachment to APTES-functionalized coverglass.

Although formaldehyde/PFA is the most commonly used fixative for synaptosome studies, it induced the clumping of synaptosomes. This was easily visible when we compared PFA-fixed synaptosomes and non-fixed mouse synaptosomes. By contrast, EGS fixation did not alter the apparent size distributions (Fig. 1a). We have also observed similar extents of PFA-induced aggregation previously with human synaptosomes^12,16^, although it was still possible to find sufficient non-clumped synaptosomes for imaging analyses. While experimenting with various synaptosome fixation and immobilization methods, we made the fortuitous discovery that resuspended synaptosomes, regardless of fixation status (none, PFA, or EGS), spontaneously adhered to glass coverslips carrying positive charges (Fig. 1b). It did not matter if the coverslip was modified with poly-lysine, bovine serum albumin, or 3-aminopropyltriethoxysilane (APTES). We chose to utilize APTES-treated glasses because the reagent was inexpensive and the coating was chemically durable.

### Zeta potential of synaptosomes

**Figure 2:**
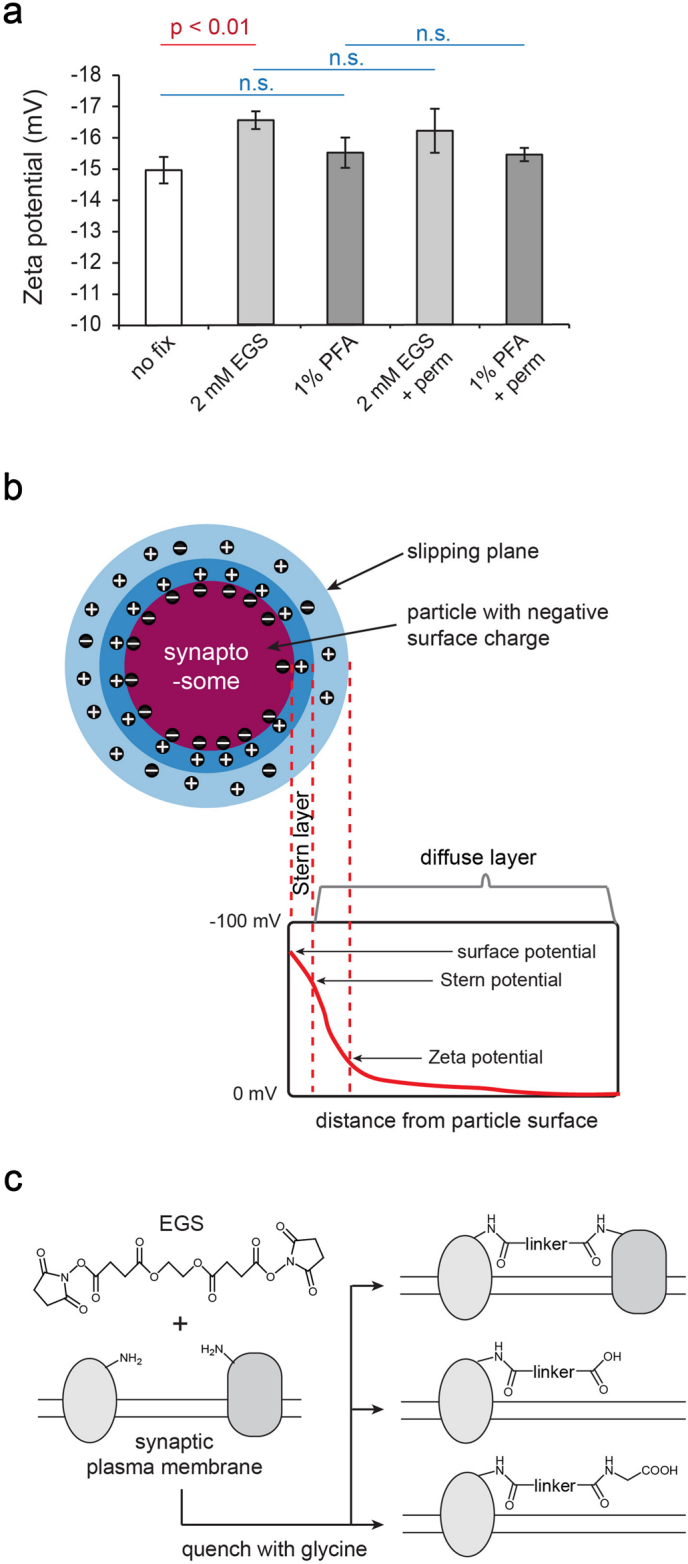
(**a**) Zeta potential measurements of synaptosomes after fixation by EGS or PFA, and permeabilized by Triton X-100 (n = 6, Welch’s t-test, n.s. = not significant). (**b**) Zeta potential is the electrical potential at the slipping plane. (**c**) Surface protein modifications following the fixation with EGS and quenching with glycine.

The spontaneous binding described above led us to hypothesize that synaptosome surfaces may be negatively charged. The surface electrical potential of synaptosome particles has not been reported before. Our measurements via dynamic light scattering found the zeta potential of synaptosomes to be − 15.0 ± 0.4 mV (mean ± standard error) at pH 7.4 (Fig. 2a). The zeta potential represents the electrical potential at the slipping plane (Fig. 2b)^38^. In comparison, zeta potentials for HeLa cells and human erythrocytes were − 19 mV and − 32 mV, respectively^39^. The source of negative charges on the synaptic surface may include acidic glycans (e.g. sialic acids), acidic protein side chains (Asp and Glu), and negatively charged phospholipids (e.g. phosphatidylserine and phosphatidylinositol)^39,40^.

For colloidal suspensions (1–1000 nm), a zeta potential of − 10 mV to − 30 mV corresponds to incipient instability, with some tendency for particle coagulation^41^. Therefore, it would be advantageous to further increase the negative charge on synaptosomes to enhance repulsion. We chose glycine as the quenching reagent for excess EGS and expected this combination to make the synaptosome surface potential more negative. As illustrated in Fig. 2c, the bifunctional EGS eliminated positive charges on surface proteins by reacting with two lysine residues. If one of the NHS esters on EGS failed to react with a lysine, subsequent quenching by glycine or auto-hydrolysis would add a negative charge. Indeed, EGS/glycine made the zeta potential significantly more negative by ~ 2 mV, but no change was caused by PFA fixation (Fig. 2a).

### Immobilization of synaptosomes for fluorescence imaging

Based on these observations, we devised a simple protocol for the fixation and immobilization of brain synaptosomes, as shown in Fig. 1b. First, synaptosomes were isolated from mouse cortical tissues using standard centrifugation methods. After treatments with EGS/glycine, synaptosomes were automatically attracted to APTES-modified glasses via electrostatic interaction. This attraction was very strong, sufficient to resist multiple rounds of washing required for immunostaining procedures, or even mild sonication. The electrostatic repulsion between EGS-fixed synaptosomes ensured good dispersion without aggregation artefacts.

**Figure 3:**
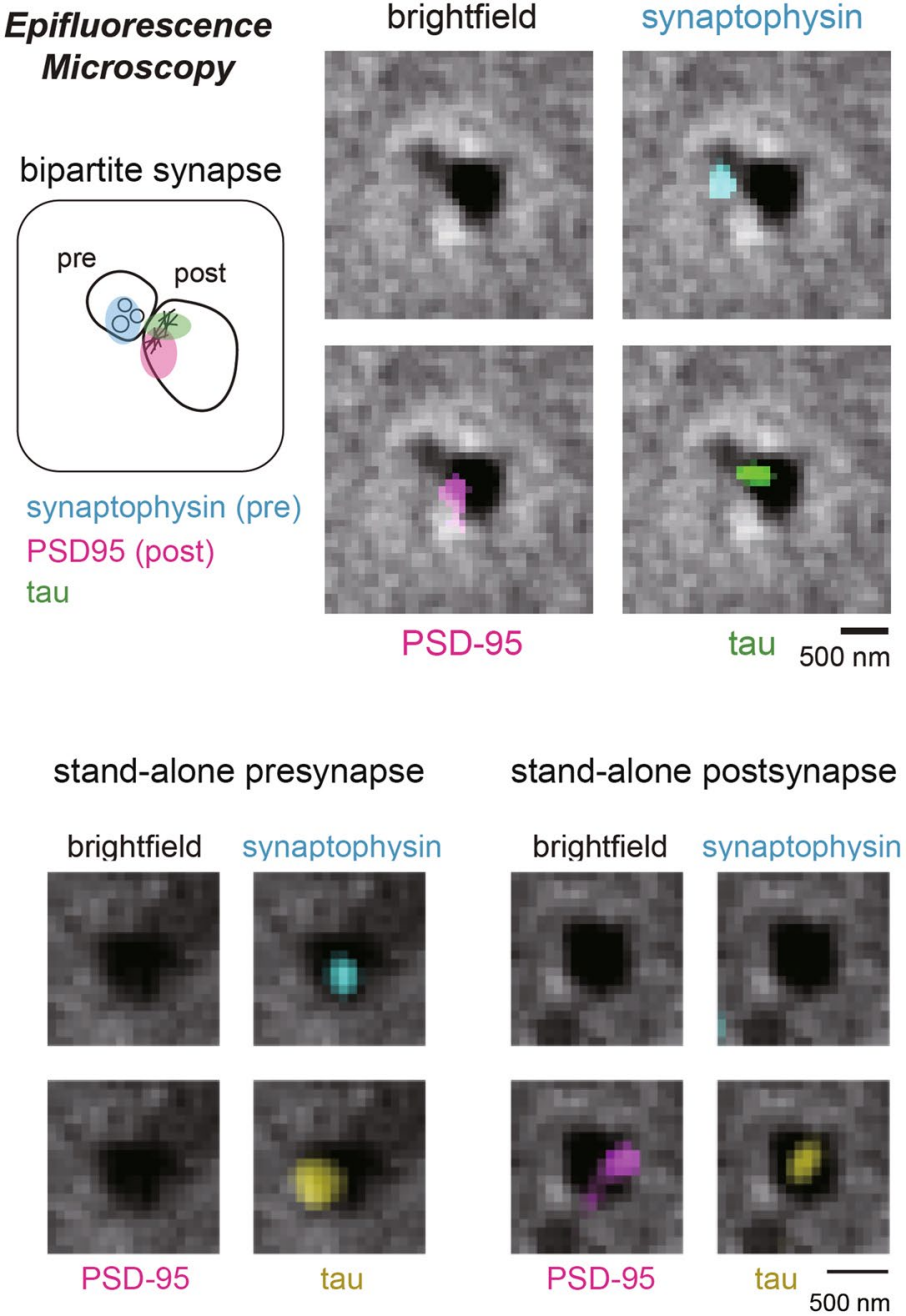
Epifluorescence images of a bipartite synapse immunostained against tau and presynaptic/postsynaptic markers. Fluorescence signals are overlaid on top of the brightfield image.

Wild-type mouse cortical synaptosomes adhered to glass coverslips were subjected to standard immunocytochemistry procedures to examine tau protein localization. We chose synaptophysin as the presynaptic marker^42^ and PSD-95 as the postsynaptic marker^43^. Under epifluorescence microscopy, tau was frequently observed at both presynaptic and postsynaptic sites (Fig. 3). Because tau is a typical axonal marker protein, its presence in presynaptic terminals (derived from *en-passant* boutons) was expected. But we also found tau to be frequently present in postsynaptic terminals (derived from mushroom spines). These results were consistent with our previous observations with human cortical synapses^12^.

### Super-resolution imaging by dSTORM

**Figure 4:**
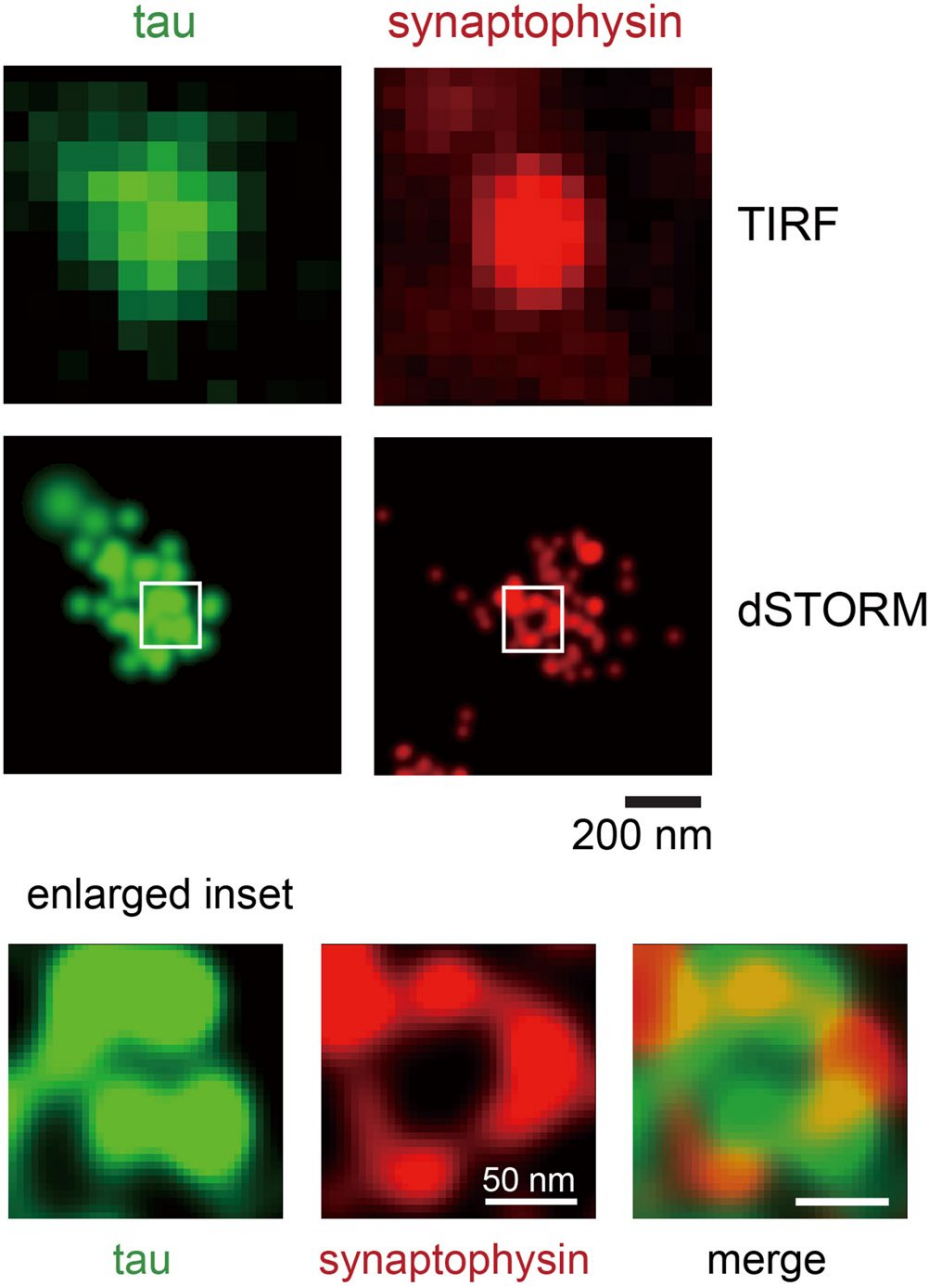
TIRF images of a presynaptic terminal containing tau protein, and the corresponding dSTORM image. Notice the overlap of tau with synaptic vesicles (marked by synaptophysin) in the enlarged insets.

The average diameter of presynaptic and postsynaptic terminals in synaptosomes is ~ 500 nm^44,45^, not much larger than the lateral resolution of diffraction-limited microscopy at ~ 200 nm. Therefore, super-resolution imaging is required for resolving protein compartmentalization within synapses^24,46^. In Fig. 4, we conducted two-color dSTORM on a total internal reflection fluorescence (TIRF) microscope (z resolution ~ 100 nm) to examine synaptophysin and tau. The subdiffraction resolution provided by dSTORM (~ 30 nm in x–y direction) allowed us to detect the ring-like clustering of synaptophysin, which likely represented synaptic vesicles docked at the active zone. Other groups have also observed similar clustering of synaptic vesicles at active zones using super-resolution imaging^24,47,48^. These results demonstrated that EGS was a mild fixative capable of preserving ultrastructural features. Importantly, we observed tau localized to synaptic vesicle clusters, which may explain why tau has been reported to be released during synaptic stimulation^11,49^. Whether tau is located inside or at the outside periphery of synaptic vesicles will need to be further investigated.

**Figure 5:**
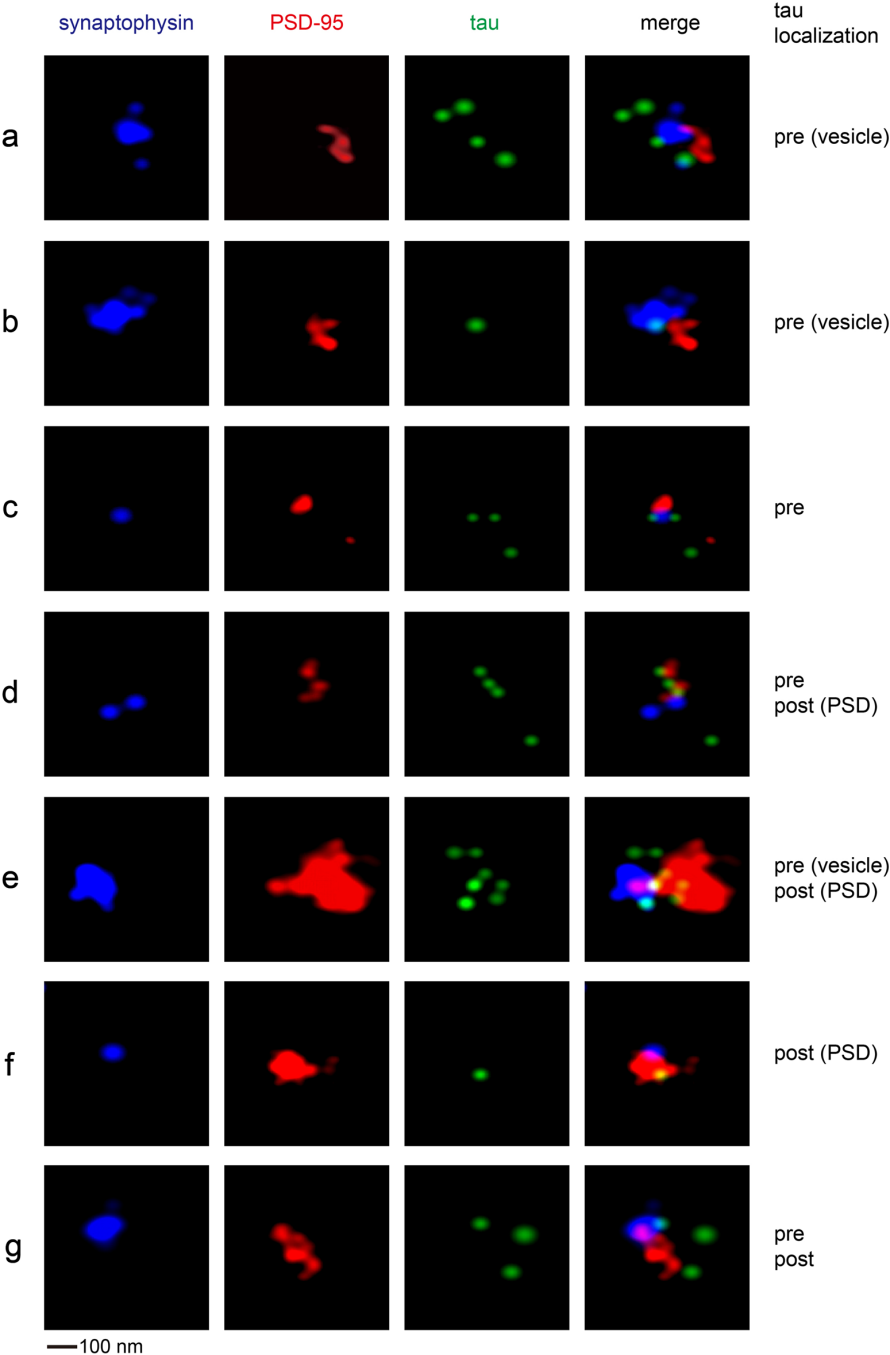
Three-channel dSTORM immunofluorescence imaging of cortical synaptosomes, against synaptophysin, PSD-95, and tau. Examples of bipartite synapses with different tau localization patterns are presented.

Next, we conducted three-color dSTORM against synaptophysin, PSD-95, and tau (Fig. 5). In this case, the lateral resolution was ~ 50 nm due to accommodations for an extra channel in our camera setup. This allowed us to putatively recognize the position of the synaptic cleft, which divided the synaptophysin-positive active zone and PSD-95-positive postsynaptic density. As expected, we observed tau protein on both sides of the synaptic cleft. The postsynaptic tau signals were often overlapping with the PSD. Low levels of tau have been previously found to co-precipitate with the PSD pellet from synaptosomes of healthy mice^50^ and human brains^12^. In summary, our dSTORM data indicate that tau protein is often associated with synaptic vesicles at the presynapse, and also with PSD at the postsynapse. It implies that normal tau protein under physiological conditions may play a role in synaptic transmission or plasticity.

## Discussion

The conventional fixative for the immunocytochemistry of synaptosomes has been formaldehyde/PFA^12,15–19^, which has the disadvantage of inducing aggregation. This artefact has impeded further progress towards super-resolution imaging or automated image analysis. Compared to formaldehyde, EGS fixation utilized in this study offers several advantages. Firstly, the crosslinking bridge moiety in EGS (12 atoms) is much longer compared to formaldehyde (1 atom), leaving more spaces between crosslinked proteins for better epitope exposure and antibody penetration^51^. Secondly, formaldehyde fixation is chemically reversible and impossible to quench completely^25,26^. In contrast, EGS fixation is easily quenched by glycine or auto-hydrolysis^52^ and forms stable, irreversible linkages. Thirdly, the more negative zeta potential imparted by EGS and the elimination of local pockets of positive charges from lysine residues may enhance electrostatic repulsions between synaptosome particles. Consequently, EGS fixation circumvents the aggregation artefacts caused by formaldehyde. EGS-fixed synaptosomes have strong affinities for APTES-functionalized coverglass, and this constitutes a reliable and simple immobilization method. Our synaptosome preparation is highly suitable for immunofluorescence studies, demonstrated by the successful imaging of tau localization using two-color and three-color dSTORM on a TIRF microscope.

Additional methods to examine ultrastructural features within synapses include immunogold-electron microscopy (EM)^53,54^ and array tomography combined with super-resolution fluorescence imaging^55–57^. Both methods can provide better z-resolution than TIRF (~ 100 nm^58^) by cutting ultrathin slices with 20–50 nm thickness. However, embedding in plastic resins is required for slice-cutting and the resin seriously impairs antibody penetration^59,60^. Therefore, the major advantage offered by our synaptosome imaging strategy is highly efficient immunolabeling in the absence of aggregation artefacts. This may allow quantitative and computer-automated analyses of protein colocalization in the future^17,61^. Our EGS fixation/electrostatic capture strategy is not only useful for imaging synaptosomes, but should be equally applicable to other subcellular fractions such as mitochondria and exosomes. Both mitochondria^62^ and exosomes^63^ also carry negative surface charges.

As proof-of-principle, we successfully imaged tau protein by two-channel and three-channel dSTORM. Despite its importance in AD, we know surprisingly little about the physiological function of tau away from the axonal microtubule^21,64^. It is very difficult to differentiate between the microtubule-related functions of tau from its synaptic roles. Normal tau proteins appear to be released via synaptic vesicles^49^ and also via exosomes^65^. Normal tau is also found at postsynaptic terminals and in a complex with PSD-95 and N-methyl-D-aspartate receptors^50,66^. Here, we provide supporting evidence that, within the subdiffraction resolution of dSTORM/TIRF, normal tau proteins are colocalized with markers of synaptic vesicles as well as PSD. This supports the notion that tau protein has physiological functions at both presynaptic and postsynaptic sites, which may partly explain its involvement in long-term depression in the hippocampus^67,68^. The synaptic compartmentalization mechanisms of tau remain somewhat elusive. Its association with synaptic vesicles could be due to direct interactions with membranes^69,70^. Its presence in dendritic spines could be related to actin-binding capacity^71,72^. Tau presence in the PSD may partly depend on its binding to Fyn kinase^73,74^. More systematic super-resolution colocalization experiments combined with mutational analyses will be required to understand the ultrastructural distribution of tau at synapses.

## Materials and methods

### Experimental animals

C57BL/6 J mice were purchased from the Laboratory Animal Center, National Taiwan University. Animals were housed with food and water available ad libitum in the animal rooms. Animal rooms were maintained at 22 ± 2 °C and were kept on a 12:12 light/dark cycle (lights on at 05:00). Male and female mice between 3 and 6 months old were used for the experiments. The minimum number of mice was used to meet the 3R principle of animal use. All animal procedures were performed according to the protocols approved by the Animal Care and Use Committee of National Taiwan University. Adequate measures were utilized to minimize potential pain or discomfort experienced by the mice used in this study. All the experiments involving animals adhered to relevant ethical guidelines as well as the ARRIVE guidelines.

### Synaptosome isolation

After mice were euthanized using isoflurane, the brain was quickly removed from the skull without perfusion, and the cerebrum was excised and transferred to a microcentrifuge tube on ice, followed by rapid freezing in liquid nitrogen, and stored at − 80 °C. Frozen mouse cortical tissue was thawed on ice. Ten volumes of ice-cold 0.32 M sucrose with 25 mM HEPES (pH 7.5), protease inhibitor cocktail (G-Biosciences), 2 mM dithiothreitol, and 5 mM EDTA were added to the tissue. The tissue was homogenized in a Potter–Elvehjem homogenizer (0.1–0.15 mm clearance) by 7 gentle up and down strokes at 170 rpm. The homogenate was spun twice at 1000×*g* for 5 min to remove nuclei and cell debris. The resulting supernatant was centrifuged at 12,000×*g* for 15 min to obtain the crude synaptosomal pellet (P2). The P2 pellet was subsequently washed with sucrose buffer, dispensed into small aliquots, and centrifuged at 12,000×*g* for 10 min. P2 pellets could be snap frozen in liquid nitrogen and stored at − 80 °C for later experiments.

### Synaptosome fixation

Each synaptosome pellet (typically from 10 to 15 mg of cortical tissue) was resuspended in ice-cold phosphate buffered saline (PBS) by gentle pipetting and passed once through a 27-gauge needle. Fixation in PBS with 1% PFA was conducted for 10 min at 4 ℃, and the reaction was quenched with 50 mM glycine pH 7.0 for 30 min at 4 ℃. Alternative fixation was conducted by adding 2.5 mM EGS (Thermo) in PBS. After 10 min of incubation, the reaction was quenched with 50 mM glycine pH 7.0 for 30 min at 4 ℃.

### Zeta potential analysis

Freshly prepared synaptosomes pellets were resuspended in PBS (pH 7.4), followed by zeta potential measurements performed with a Zetasizer Nano-ZS (Malvern Instruments) at 25 ℃. Statistical analysis for Welch’s t-test was computed using MS Excel.

### Glass modification with APTES

Glass-bottom dishes (with unmodified coverslip, MatTek) were rinsed twice in 95% ethanol, and functionalized by 1% APTES (Sigma) (v/v) for 20 min at 60 ℃. After functionalization, the dishes were rinsed in water three times and dried at 60 ℃ for 20 min.

### Immunofluorescence staining

APTES-coated glass-bottom dishes were used for synaptosome immunolabeling and imaging. Suspensions of EGS-fixed synaptosomes were placed over the glass-bottom dish for 1 h to facilitate immobilization. Captured synaptosomes were rinsed twice with PBS, and permeabilized with 0.05% high-purity Triton X-100 (Thermo) in PBS for 10 min, followed by two more washes with PBS (10 min each). These dishes were incubated for 30 min at 4℃ with a blocking buffer that contains 4% normal donkey serum (Millipore) and 50% SEA block buffer (Thermo) in PBS. Primary antibodies were diluted in blocking buffer and incubated for 90 min at room temperature, followed by three washes with PBS. Secondary antibodies were diluted in blocking buffer and incubated for 45 min at room temperature, followed by three washes with PBS.

### Epifluorescence microscopy and image analysis

Primary antibodies for immunostaining included mouse anti-synaptophysin (GeneTex GTX38985, RRID: AB_11170870, 1:200), goat anti-PSD-95 (GeneTex GTX88388, AB_10722757, 1:100), and rabbit anti-Tau (Dako A0024, AB_10013724, 1:400). Fluorescent secondary donkey antibodies were purchased form Jackson Immunoresearch, including anti-mouse IgG Alexa 488 (1:100), anti-goat IgG Alexa 647 (1:100) and anti-rabbit IgG Cy3 (1:100). The dishes were mounted with Prolong Gold Antifade reagent (Invitrogen). Images were acquired on a Zeiss AxioImager Z1 epifluorescence microscope equipped with a 63 × oil immersion objective (numerical aperture = 1.40).

Images were deconvolved with the Iterative Deconvolution plugin (by Bob Dougherty, OptiNav Inc.) in ImageJ software (version 1.44)^75^. This 2D deconvolution program required a point spread function (PSF) generated by Diffraction Limit PSF plugin (Bob Dougherty, OptiNav Inc.). For the brightfield image we used a 400 nm PSF; for green fluorescence channel a 509 nm PSF; for red channel a 550 nm PSF; for far red channel a 650 nm PSF. The optimal iteration number was empirically determined to be 12 for brightfield images and 16 for fluorescence images, with the LP filter diameter set at 1.5 pixels. After deconvolution, brightfield and fluorescence images were overlaid in ImageJ (using hyperstacks) and protein colocalization was determined by manual inspection.

### TIRF and dSTORM

Primary antibodies for immunostaining included guinea pig anti-synaptophysin (Synaptic Systems #101004, AB_1210382, 1:50), goat anti-PSD-95 (1:30), mouse anti-tau (Tau-5, Thermo MA5-12808, AB_10980631, 1:50), and mouse anti-tau DA9 (1:50, gift of Peter Davies, Albert Einstein College of Medicine). Fluorescent secondary donkey antibodies were purchased form Jackson Immunoresearch, including anti-guinea pig IgG Alexa 647 (1:20), anti-goat IgG Alexa 594 (1:20) and anti-mouse IgG Alexa 488 (1:20).

The dSTORM system consisted of TIRF microscope with a scientific complementary metal–oxide–semiconductor (sCMOS) camera (Andor) mounted on an inverted fluorescence microscope (IX71, Olympus), for which the excitation sources are a 473 nm solid state laser, a 561 nm solid state laser and a 637 nm solid state laser (CNI). We used an oil-immersion TIRF objective (100X, Olympus) with a 1.49 numerical aperture, a dichroic beamsplitter (Di01-R405/488/561/635, Semrock), and a filter wheel with three emission filters (FF01-525/45-25, FF01-593/46-25, and FF01-692/40-25, Semrock). The excitation power was 0.7, 1.2, and 1.4 kW cm^−2^ for Alexa 488, Alexa 594, and Alexa 647 stained specimens, respectively. To achieve fluorescence switching of fluorophores, phosphate buffered saline with 10 mM of β-mercaptoethanol was added into the sample before capturing images. An sCMOS camera was used to record at 20 frames/second the blinking fluorescence images in dSTORM. The three fluorophores were imaged sequentially with an order from longer to shorter emission wavelengths. The localization and reconstruction of dSTORM images were performed by the MATLAB program using the sCMOS camera–specific single-molecule localization algorithm, which had been reported previously^76,77^.
